# The effect of meteorological factors on severe fever with thrombocytopenia syndrome: Evidence from 34 Chinese cities

**DOI:** 10.1016/j.onehlt.2025.101295

**Published:** 2025-12-11

**Authors:** Guangju Mo, Xiyuan Huo, Meshack Kipkogei Biwott, Nan Chang, Haoqiang Ji, Lianfang Feng, Huaiping Zhu, Qiyong Liu

**Affiliations:** a School of Public Health, Shandong Second Medical University, Weifang, Shandong Province, China; b National Key Laboratory of Intelligent Tracking and Forecasting for Infectious Diseases, National Institute for Communicable Disease Control and Prevention, Chinese Center for Disease Control and Prevention; WHO Collaborating Centre for Vector Surveillance and Management, Beijing, China; c Weifang Center for Disease Control and Prevention, Weifang, Shandong Province, China; d School of Public Health, Nanjing Medical University, Nanjing, Jiangsu Province, China; e School of Public Health, Shandong University, Jinan, Shandong Province, China; f LAMPS and CDM, Department of Mathematics and Statistics, York University, Toronto, Canada

**Keywords:** Severe fever with thrombocytopenia syndrome, Emerging infectious disease, Meteorological factors, DLNM, Meta-analysis

## Abstract

**Background:**

Severe fever with thrombocytopenia syndrome (SFTS) is a climate-sensitive infectious disease, and its spatial distribution has been expanding in recent years. This study aimed to investigate the influence of meteorological factors on SFTS incidence.

**Methods:**

Data on SFTS was extracted from the Infectious Disease Surveillance Report Management System from January 1, 2011 to December 31, 2023. A two-stage hierarchical analytical framework was employed in this study. First, a distributed lag nonlinear model was utilized to characterize the nonlinear exposure-response relationships between meteorological factors and the incidence of SFTS at the municipal level. Second, a multivariate meta-analysis was conducted to synthesize city-specific effect estimates, with explicit adjustment for inter-regional heterogeneity.

**Results:**

From 2011 to 2023, 34 cities with cumulative cases ≥100 were included in the final analysis, which accounted for 94.59 % of the total SFTS cases during the same period in mainland China. The incidence risk of SFTS was positively correlated with temperature, relative humidity, precipitation, normalized difference vegetation index, and land cover, but negatively correlated with atmospheric pressure. The exposure-response relationship between average temperature and SFTS risk exhibited a single peak at 24.70 °C (*RR* = 2.78, 95 % *CI*: 1.14–6.79). Stratified analysis revealed the highest temperature-related risk in Eastern China at 27.50 °C (*RR* = 9.85, 95 % *CI*: 1.87–51.76), which was significantly elevated compared to central and northeastern regions. Regional variability was also observed for precipitation: the overall minimum risk occurred at 15.30 mm (*RR* = 0.49, *95 % CI*: 0.24–0.98), whereas the risk nadir in Eastern China was at 16.02 mm monthly precipitation (*RR* = 0.29, *95 % CI*: 0.10–0.80).

**Conclusions:**

This study demonstrates that temperature and precipitation significantly influence SFTS incidence, with effects lagging consistently by 1–2 months. These findings can be integrated into China's Smart Multi-Point Surveillance System by incorporating region-specific meteorological thresholds to trigger early warnings. The system could then activate targeted interventions, such as tick control measures, accounting for the observed 1–2 month lag between climatic conditions and disease occurrence. Such climate-adaptive approaches would enhance the precision and timeliness of SFTS prevention and control efforts nationwide.

## Highlights

• Systematically evaluated the relationship between meteorological factors and the risk of SFTS incidence at the national scale
• Temperature and precipitation are key meteorological factors influencing the risk of SFTS incidence
• The influence of meteorological factors on SFTS incidence risk exhibits spatial heterogeneity.

## 1 Introduction

Severe Fever with Thrombocytopenia Syndrome (SFTS) is an emerging infectious disease caused by a bunyavirus, initially identified as the severe fever with thrombocytopenia syndrome virus (SFTSV) and later renamed Dabie bandavirus (DBV) [[1], [2], [3]]. In recent years, SFTS cases have been reported not only in East Asian countries such as Japan [4], Korea [5,6], and Southeast Asian countries like Thailand [7,8], but also in other regions, including Kenya [9], where the risk of SFTS has recently been confirmed, with potential for further spread across African counties. Clinically, SFTS is characterized by high fever, thrombocytopenia, leukocytopenia, and multi-organ impairment, with severe cases potentially progressing to fatal outcomes [10]. The transmission of this disease mainly occurs through *Haemaphysalis longicornis* (*H. longicornis*) bites and close contact with infected individuals, making its epidemiology closely related to environmental factors [[11], [12], [13]].

Meteorological factors, such as temperature, precipitation, and humidity, play a crucial role in regulating the life history and behavior patterns of vector species, thereby affecting the dynamics of disease transmission [7]. Suitable temperature and humidity promote tick development and host-seeking behavior. Precipitation demonstrates dual effects as moderate rainfall creates favorable tick habitats while heavy rains may cause physical displacement of populations. These interconnected elements together form an environmental regulatory system that determines tick population dynamics and viral transmission potential [14]. Due to significant climatic differences across various regions in China, the epidemic characteristics of SFTS may vary accordingly [15]. Therefore, investigating the associations between meteorological factors and SFTS incidence can yield critical scientific evidence to inform the prevention and control strategies for this disease. Meanwhile, environmental determinants including Normalized Difference Vegetation Index (NDVI) and land cover types directly modulate SFTS risk by altering vector habitat suitability. Elevated NDVI values indicating dense vegetation provide optimal tick microhabitats and support wildlife host populations, establishing a “vegetation-host-tick” transmission cycle. Land use patterns create spatial heterogeneity, with agricultural-forest ecotones representing high-risk zones due to frequent human activity, while urbanized areas may fragment natural habitats yet maintain transmission hotspots in green spaces [16,17]. Concurrently, socioeconomic factors such as urbanization rate and GDP per capita indirectly influence transmission dynamics through modified human-environment interactions, where habitat encroachment drives wildlife into human settlements, and improved public health infrastructure enhances disease surveillance [18]. These interconnected environmental and social drivers collectively construct an “ecological-social” complex system governing SFTS spread.

Previous studies have found a nonlinear relationship between meteorological factors and the incidence of SFTS in a single city or province, with some lag effect [[19], [20], [21], [22], [23]]. This highlights the growing importance of assessing the impact of meteorological factors on the spread and transmission of SFTS. However, the study areas were limited to a single area in available research and did not account for the variations and differences in geographical locations [24]. This national-scale investigation provides novel insights by quantifying region-specific exposure-response patterns for key meteorological factors, systematically assessing how geographic and ecological variables modify these relationships.

In recent years, two-stage models constructed by distributed lag nonlinear model (DLNM) and multivariate meta-analysis have been widely used to evaluate the association between meteorological factors and diseases, and the results are highly informative [[25], [26], [27]]. This study employed a two-stage analytical framework to systematically examine the exposure-response relationships between meteorological determinants and SFTS incidence across 34 Chinese cities. Recognizing China's substantial climatic and environmental variations across regions, this study systematically accounts for geographical heterogeneity through multivariate meta-analysis techniques. By integrating data from diverse ecological zones, our multicenter meteorological-epidemiological framework not only enhances the generalizability of findings but also enables the development of spatially tailored prevention strategies. This approach fundamentally advances our understanding of SFTS climate sensitivity at national scale, while providing actionable evidence for building climate-adaptive early warning systems - a critical step toward precision public health in infectious disease control.

## 2 Methods

### 2.1 Study area and data sources

The study sites covered 34 cities in seven provinces in mainland China. Cities with a cumulative number of cases below 100 from 2011 to 2023 were excluded to ensure model robustness, as smaller samples may introduce high variance in estimates [28]. Similar thresholds are recommended in infectious disease mapping [25,29]. The included cities were further categorized into three regions based on different regional characteristics: eastern, central and northeastern.

### 2.2 Data on severe fever with thrombocytopenia syndrome cases

Since 2010, SFTS has been reported online according to the requirements of class B notifiable infectious diseases in China. The daily number of SFTS cases was obtained from the infectious disease control and prevention information system of the Chinese Center for Disease Control and Prevention. All healthcare institutions were required to report clinically diagnosed and laboratory-confirmed SFTS cases within 24 h of identification, strictly adhering to the workflow of China's Infectious Disease Network Direct Reporting System. Only clinically diagnosed cases and confirmed cases were included in this study, and suspected cases were excluded (eFigure 1 in Supplementary file). The diagnostic criteria of the cases were based on the diagnosis and treatment program of SFTS (2010 edition) (eMethods in Supplementary file). This study is an epidemiological survey aimed at analyzing the trends of incidence. According to relevant ethical guidelines, since this study does not involve direct intervention with participants or potential risks, it does not require ethical approval. This study adhered to the Strengthening the Reporting of Observational Studies in Epidemiology (STROBE) guidelines for reporting observational research (eTable 1 in Supplementary file).

### 2.3 Data on meteorological, environmental, and social factors

Daily meteorological data were extracted from the National Meteorological Information Centre (https://data.cma.cn/). For each city, we collected average temperature (TEM_Avg) (°C), average relative humidity (RHU_Avg) (%), average atmospheric pressure (PRS_Avg) (hPa), average wind speed (WIN_S_Avg) (m/s), and precipitation (PRE) (mm), from 1 January 2011 to 31 December 2023. Monthly average temperature, relative humidity, atmospheric pressure, wind speed, and precipitation are averaged for each month. NDVI was obtained from the Resource and Environmental Sciences Data Platform (https://www.resdc.cn/DOI/ doi.aspx?DOIid = 50). Land cover data was sourced from the annual China Land Cover Dataset (CLCD), which was generated annually on the Google Earth Engine (GEE) platform using Landsat satellite data [30]. Data on per capita GDP and Urbanization rate were derived from the statistical yearbook of each city.

### 2.4 Statistics analysis

A descriptive statistical analysis was performed on the SFTS case data and meteorological variables. Simultaneously, Spearman correlation analysis and multicollinearity diagnostics were conducted for climatic, environmental, and socioeconomic factors. Variables with correlation coefficients <0.7 and variance inflation factors (VIF) ≤ 5 were considered control variables for subsequent analysis [31]. Next, we implemented a hierarchical two-stage analytical framework to quantify the nonlinear exposure-lag-response associations between meteorological determinants and SFTS incidence across spatial scales. In the first stage, a DLNM for individual cities was established based on Quasi-Poisson regression to derive the relative risk (*RR*) of SFTS associated with each meteorological factor for specific cities. The basic model is as follows:

$$\log\left[E\left(Y_{t}\right)\right]=\alpha +\mathrm{cb}\left(X_{t},1\right)+\mathrm{ns}\left(\text{meteorological factors},\mathrm{df}\right)+\mathrm{ns}\left(\text{time},\mathrm{df}\times \text{year}\right)+\text{season}+\text{Control variables},$$

The degrees of freedom (*df*) and number of lag months were chosen based on previous studies and the Akaike information criterion (AIC) [32,33]. where *Y*_*t*_ is the number of SFTS cases in month *t*, *α* is the intercept, *cb (X*_*t*_*, 1)* is the cross base matrix of meteorological factors, l is the maximum number of lag months, which was set to 2 based on three key considerations. First, it accounts for the complete transmission cycle, including the 1–2 month development of *H. longicornis* ticks and their subsequent host-seeking activity, despite the shorter 7–14 days human incubation period. Second, this duration aligns with epidemiological observations showing SFTS case peaks typically occur 2–4 months after favorable weather conditions [18,34]. Third, statistical analysis confirmed the 2-month lag provides optimal model fit (*AIC* = 1441.295), outperforming longer lag periods (3-month:1487.19, 4-month:1599.29). This integrated approach ensures our model accurately reflects both the biological transmission dynamics and observed disease patterns. The parameter *argvar* controls the relationship between meteorological factors and the incidence of SFTS, with the degrees of freedom set to 3 and the basis function using a *quadratic B-spline*, while the reference value is the median value of meteorological factors. The parameter *arglag* controls the lag effects, with the degrees of freedom set to 3 and using a natural cubic spline function. *ns* (*meteorological factors, df*) is the natural cubic spline function of other meteorological factors, which has a degree of freedom of 3. *time* was used to control the long-term trend, which has a degree of freedom of 3/year. *Season* was incorporated into the model to adjust for seasonal variations in the time-series analysis.

In the second stage, a multivariate meta-analysis was conducted to synthesize the data and determine the overall exposure-response relationship. *RR* and 95 % confidence intervals (*CI*) were calculated using the median values of each meteorological factor. *Cochran's Q* test and *I*^*2*^ statistic were used to assess heterogeneity [35]. Between-study heterogeneity was quantified using the *I*^*2*^ statistic: 25–49 % (low), 50–74 % (medium), and ≥ 75 % (high). The model selection followed a double-criteria framework: a fixed-effect model was prespecified when *I*^*2*^ < 50 % and *Cochran's Q*-test *P* > 0.10, and a random-effect model was used when *I*^*2*^ ≥ 50 % or *Q*-test *P* ≤ 0.10. To address geographic heterogeneity, we conducted stratified analyses across three macro-regions (Eastern, Central, and Northeastern China) as per the National Bureau of Statistics classification system. In addition, we explored the lagged effects of different quartiles (P_5_ and P_95_) as specific values of each meteorological factor with the incidence of SFTS, where P_5_ is defined as a low level and P_95_ as a high level. Finally, we determined the robustness of our findings by varying the time trend (2–4 *df* per year), and lag time (2–4 months) for sensitivity analysis. The analysis was performed using *R* (4.5.1) and the software packages “dlnm” and “mvmeta”.

## 3 Results

### 3.1 Epidemiological characteristics of SFTS

**Fig. 1:**
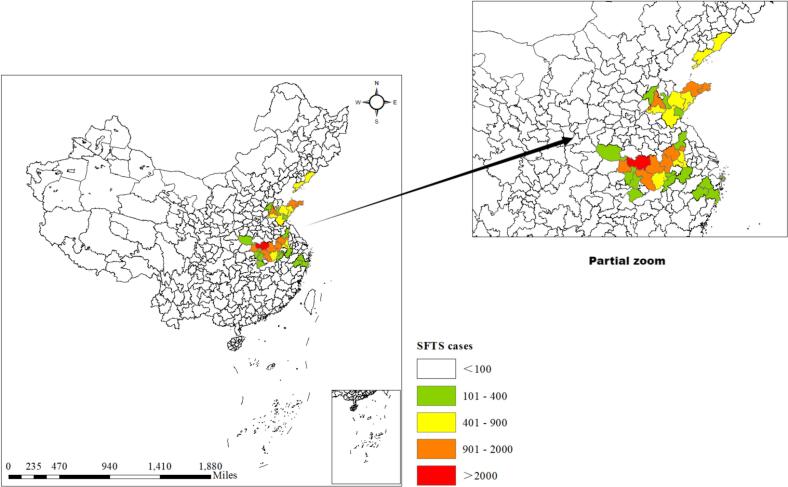
Spatial distribution of SFTS cases in 34 cities, 2011–2023.

**Fig. 2:**
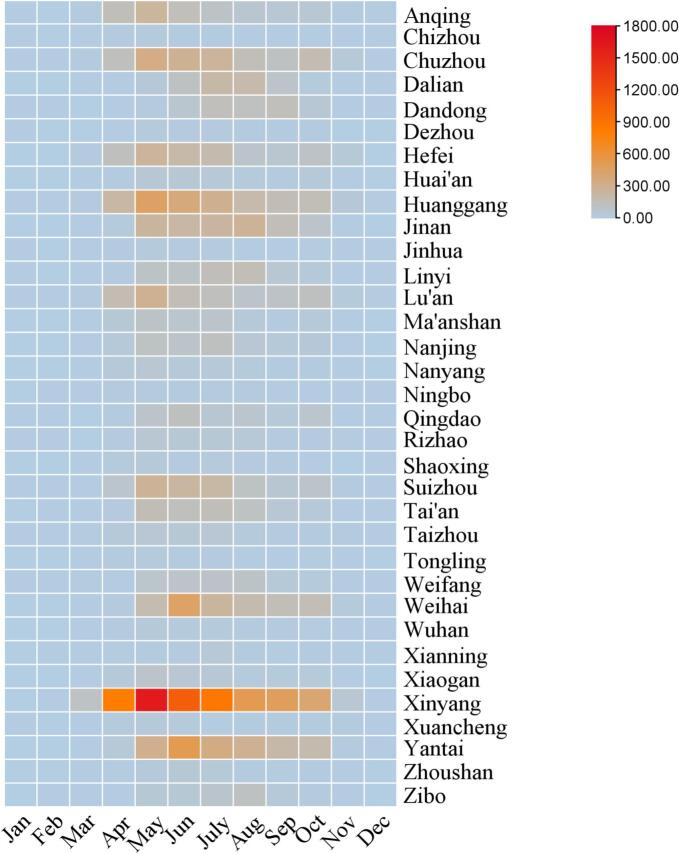
Monthly distribution of SFTS cases in 34 cities, 2011–2023.

From 2011 to 2023, 34 cities with the highest number of cases are mainly concentrated in Shandong, Henan, Anhui, Hubei, Liaoning, Zhejiang, and Jiangsu provinces (Fig. 1). A total of 25, 901 SFTS cases were reported in the selected cities, accounting for 94.59 % (25, 901/27, 382) of the total number of cases nationwide. In terms of temporal distribution, the incidence of SFTS shows a distinct seasonal distribution, mainly concentrated between April and October. Typically, the peak occurs in May and June (Fig. 2).

### 3.2 Correlation analysis between SFTS incidence, meteorological, environmental, and social factors

Statistical data on total SFTS case numbers and monthly meteorological variables for the 34 cities from 2011 to 2023 were shown in eTable2 in Supplementary file. There was notable variability in the monthly averages of different meteorological factors among the cities. The basic characteristics of each meteorological factor were shown in eTable3 in Supplementary file.

**Fig. 3:**
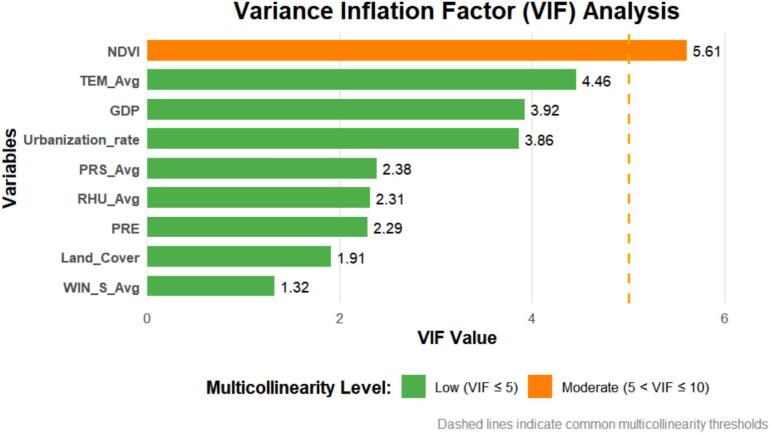
Variance inflation factor for each variables.

The Spearman correlation analysis indicated that SFTS cases are positively correlated with the monthly average temperature (*r* = 0.59), monthly average relative humidity (*r* = 0.20), monthly average precipitation (*r* = 0.38), monthly NDVI (*r* = 0.48), and land cover (*r* = 0.17), which are statistically significant (*P* < 0.05). It is negatively correlated with the monthly average atmospheric pressure (*r* = −0.37, *P* < 0.001). However, the correlation with the average wind speed, per capita GDP and urbanization rate are not statistically significant (*P* > 0.05). Of particular ecological significance, our analyses showed a strong correlation between monthly NDVI and meteorological factors (VIF > 5), particularly with monthly average temperature, suggesting a closely coupled dynamic relationship between vegetation and climate. While this multicollinearity necessitated the exclusion of NDVI from final models to ensure robust parameter estimates, its ecological relevance should be acknowledged in interpreting climate-SFTS relationships. The observed NDVI-temperature association implies that vegetation may mediate some climatic effects on tick habitats, and thus the retained temperature effects in our models may partially reflect this underlying vegetation-climate interaction. This methodological approach prioritizes model stability while recognizing the complex, interdependent nature of environmental drivers in SFTS transmission ecology (Fig. 3, eFigure 2 in Supplementary file).

### 3.3 Effect of meteorological factors on the incidence of SFTS

Based on the results of correlation analysis, four meteorological factors, namely, monthly average temperature, monthly average relative humidity, monthly average atmospheric pressure, and monthly average precipitation, were selected as study variables, with land cover serving as the control variable. When one of these meteorological factors was used as a study variable in the establishment of the DLNM model, the other meteorological factors were included in the model as confounding factors for control.

**Fig. 4:**
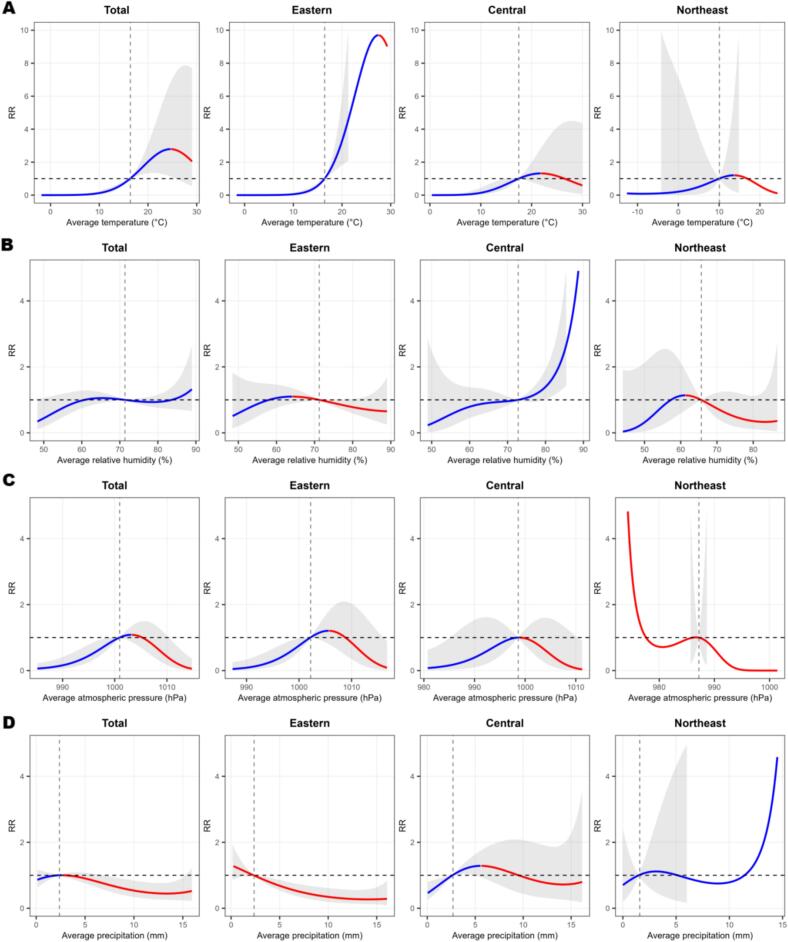
Cumulative effects of four meteorological factors (A temperature, B relative humidity, C atmospheric pressure, and D precipitation) on SFTS incidence at total and regional levels. Blue solid lines: rising phase; Red solid lines: falling phase; Dashed lines: median value of meteorological factors (vertical) and RR = 1 (horizontal). (For interpretation of the references to colour in this figure legend, the reader is referred to the web version of this article.)

Fig. 4 displayed the overall cumulative exposure-response relationships, with meteorological determinants parameterized using median values as reference exposures at total and regional levels. Regionally stratified analyses for eastern, central, and northeastern China reveal geographical heterogeneity in the climate-health association. Fig. 4A illustrated the cumulative effect of temperature on SFTS incidence risk. At the total level, monthly average temperature exhibited an inverted U-shaped relationship with SFTS incidence. The multivariate *Cochran Q-test* for heterogeneity: *Q* = 81.722, *P* = 0.896, *I*^*2*^ = 1.0 %, a fixed-effects model was selected. The exposure-response relationship peaked at 24.70 °C (*RR* = 2.78, 95 % *CI:* 1.14–6.79). At the regional level, the eastern China exhibited a higher peak SFTS risk (27.50 °C, *RR* = 9.85, 95 % *CI*: 1.87–51.76) and a narrower temperature range; Central regions exhibited a flatter SFTS risk curve (21.80 °C, *RR* = 1.31, 95 % *CI*: 0.62–2.78) with a broader temperature adaptation range; In the Northeast China, the average temperature at which SFTS risk peaks was significantly lower than in other regions (13.70 °C, *RR* = 1.20, 95 % *CI*: 0.25–5.73). Relative humidity showed a subtle U-shaped association with SFTS risk nationally (Fig. 4B), with *RR* marginally elevated at both low (<50 %) and high (>80 %) relative humidity, though 95 % *CIs* overlapped with the null value (*RR* = 1). Regionally, the trend was most evident in Central China, where *RR* increased to 2.0 under high humidity (>85 %) with a narrower *CI*, aligning with the region's dense vegetation that retains moisture for tick survival. In contrast, East China and Northeast China exhibited a pattern of moderate increase followed by decline. These findings suggest humidity may act as a context-dependent modifier, with local ecological conditions (e.g., vegetation cover) shaping its impact on tick activity. In general, the impact of atmospheric pressure changes on SFTS incidence risk presented a trend of increasing and then decreasing. When atmospheric pressure values ranged between 998.90 and 1010.20 hPa, the SFTS incidence risk value exceeded 1. However, in Northeast China, atmospheric pressure and SFTS risk follow an “extremely low pressure, high risk” pattern, contrasting with the single-peak pattern observed in other regions (Fig. 4C). The exposure-response relationship between precipitation and SFTS incidence exhibits regional heterogeneity (Fig. 4D). The minimum risk (*RR* = 0.49, 95 % *CI*: 0.24–0.98) occurs at 15.30 mm precipitation at total level, while in eastern China, the risk nadir (*RR* = 0.29, 95 % *CI*: 0.10–0.80) corresponds to a monthly precipitation threshold of 16.02 mm. In Northeast China, precipitation intensity significantly increases during heavy precipitation events, contrasting sharply with other regions. This suggests that concentrated summer downpours in the Northeast may drive ticks from vegetation into human activity areas—such as farmlands and residential zones—thereby creating a “post-rainstorm risk peak”.

P_5_ and P_95_ of each meteorological factor were selected as specific values to analyze their lagged effects on the incidence of SFTS (eTable 3 in Supplementary file). The lagged effect of temperature on the incidence of SFTS tended to increase at a low temperature of 0.01 °C, with a significant protective effect at a lag of 0–1.5 months. At high temperatures of 28.22 °C, the lag effect distribution curve of temperature on SFTS incidence exhibits a significantly downward trend (eFigure 3 in Supplementary file). At lower relative humidity (50.47 %), the lag effect on SFTS incidence shows an upward trend. In contrast, at higher relative humidity (84.04 %), a negative correlation is observed between lag time (0–2 months) and *RR* (eFigure 4 in Supplementary file). The highest risk value was observed when monthly average atmospheric pressure reached its 5th percentile (975.36 hPa), with a gradual attenuation of effect observed across the 0–2 month lag period; when at the 95th percentile (1021.40 hPa), the *RR* continuously increased over the same lag period (eFigure 5 in Supplement file). Finally, at both lower and higher percentiles, the lag effect of precipitation shows a negative correlation with the incidence risk of SFTS (eFigure 6 in Supplementary file).

**Fig. 5:**
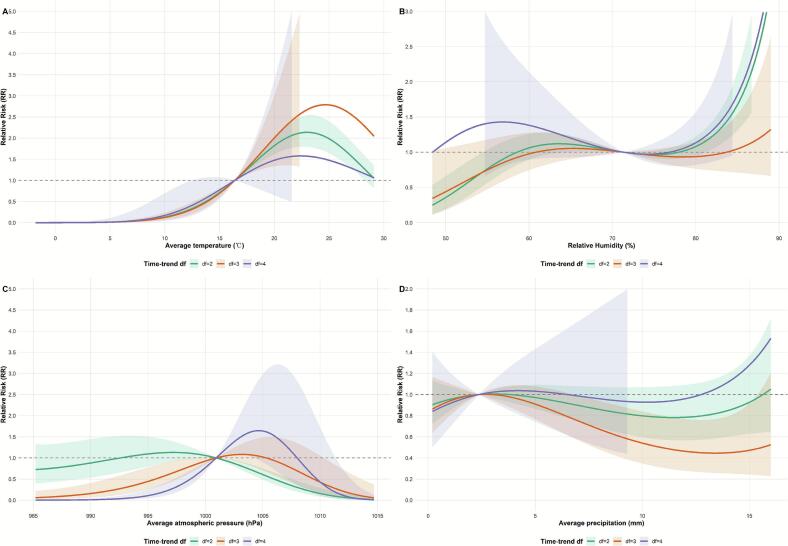
Sensitivity analysis results: changing time trend degrees of freedom (*df*). Note: (A) temperature, (B) relative humidity, (C) atmospheric pressure, and (D) precipitation, Shaded areas represent 95 % confidence intervals.

**Fig. 6:**
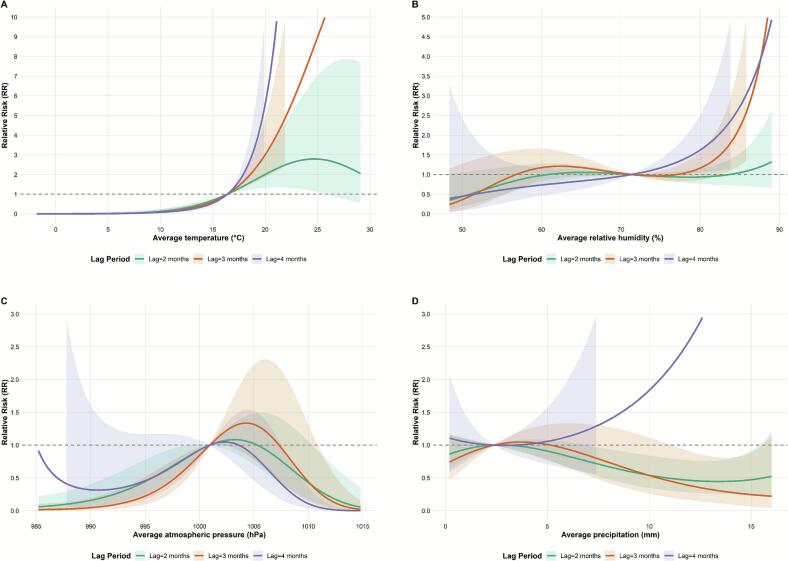
Sensitivity analysis results: changing the lag period. Note: (A) temperature, (B) relative humidity, (C) atmospheric pressure, and (D) precipitation, Shaded areas represent 95 % confidence intervals.

In the sensitivity analysis, as shown in Fig. 5, Fig. 6, the results of the cumulative effect of each meteorological factor on the risk of incidence of SFTS were more stable after varying the different time-trend degrees of freedom and lag periods.

## 4 Discussion

In this study, the relationship between meteorological factors and the incidence of SFTS was systematically analyzed using a two-stage model. This nationwide study encompassed 34 SFTS endemic areas, capturing 25, 901 SFTS cases that accounted for 94.59 % (25, 901/27, 382) of total national notifications during the study period, demonstrating strong epidemiological representativeness and comprehensive national coverage of high-risk regions.

The prevalence trend of SFTS from 2011 to 2023 shows that there are more cities with high prevalence of SFTS than in the past. In addition to Eastern and Central China, Northeastern China is also a high prevalence area for SFTS [21,[36], [37], [38], [39], [40], [41]]. The number of high-incidence cases in these areas is closely related to their ecological and climatic conditions and the activity patterns of their inhabitants [19,[42], [43], [44]]. This study reveals the complex interaction mechanism between meteorological factors and SFTS transmission by integrating climate epidemiology and nonlinear exposure-response modeling.

The findings reveal a biphasic temperature-dependent transmission pattern, characterized by an inverted U-shaped relationship with a peak risk at 24.7 °C. Development of *H. longicornis* progresses through four distinct life stages: egg, larva, nymph, and adult. Except for the egg stage, each stage is obligatorily hematophagous, requiring a blood meal to advance to the next phase, with all exhibiting temperature-dependent phenological patterns. During late winter or early spring, nymphal activity resumes in response to rising temperatures and photoperiod changes, triggering host-seeking behavior [[45], [46], [47], [48]]. Laboratory studies indicate an optimal temperature range of 18–35 °C for *H. longicornis*, with a documented survival threshold between 12 and 40 °C. Thermal stress becomes lethal above 40 °C, while temperatures below 12 °C suppress or halt host-seeking activity [[49], [50], [51]]. Notably, field observations from South Korea suggest broader climatic adaptability, with survival recorded across 5.7–42.1 °C, highlighting the species' ecological plasticity [52]. In addition to, moderate rainfall reduces tick habitat humidity and disrupts host-vector contact, whereas drought or heavy rainfall promotes host-seeking behavior and habitat reconstruction, underscoring the nonlinear ecological responses to hydrological changes [22]. Furthermore, our study found that relative humidity, atmospheric pressure, and precipitation were not significantly associated when the cumulative effect of risk of SFTS incidence peaked. The observed attenuation of individual meteorological effects may stem from multicollinearity among climatic variables. Temperature, relative humidity, and precipitation are ecologically coupled. For example, higher temperatures accelerate evaporation, reducing surface humidity, while heavy rainfall simultaneously increases ambient humidity and cools the environment. This creates a “see-saw effect” where variables mask each other's independent impacts in regression models. Our VIF analysis confirmed moderate collinearity, consistent with previous studies [53,54]. In addition, ticks exhibit compensatory behaviors—e.g., during droughts, they seek microhabitats with residual moisture (e.g., leaf litter), offsetting the apparent influence of relative humidity. This behavioral plasticity dilutes statistical signals in aggregated models [55,56].

Meteorological factors may influence SFTS transmission not only indirectly by modulating tick vector populations but also directly through altering human outdoor behavioral patterns, thereby independently amplifying exposure risks. For instance, during mild and rainy spring seasons (18–25 °C), optimal temperature and humidity simultaneously boost tick activity (nymphal host-seeking peaks) and agricultural practices (e.g., tea picking, farming), leading to a synergistic increase in human-tick encounters [57]. Conversely, extreme heat or prolonged rainfall might suppress tick questing behavior but could shift farming activities to dawn or dusk—coinciding with peak tick diurnal rhythms—paradoxically elevating exposure risks. Furthermore, seasonal surges in recreational activities (e.g., hiking) overlapping with critical tick life stages may explain nonlinear spatiotemporal patterns in SFTS incidence [57,58].

The pronounced spatial heterogeneity in climate-disease associations underscores the necessity of geographic stratification in SFTS risk modeling. This is exemplified by the divergent temperature thresholds between eastern China (27.50 °C, *RR* = 9.85) and northeastern regions (13.70 °C, *RR* = 1.20). The heightened temperature sensitivity observed in eastern China likely results from the synergistic interplay of three key factors: (1) Population density: Eastern regions (e.g., Shandong, Jiangsu) exhibit higher rural population densities [59], which significantly increase human-tick contact during agricultural activities; (2) Agroforestry landscapes: The mixed cropping systems and fragmented woodlands characteristic of this region create ecological “funnels” where ticks, wildlife hosts (e.g., hedgehogs, rodents), and humans frequently intersect [2,60,61]. Notably, temperature-driven peaks in tick activity (20–28 °C) coincide with rice planting seasons (May–June), further amplifying exposure risks; (3) Vegetation-mediated microclimates: The dense vegetation cover in eastern China buffers extreme temperatures, maintaining stable tick habitats, while northeastern forests experience greater thermal variability, reducing tick survival consistency. While all three factors synergistically elevate risk, population density and agricultural timing are the predominant determinants of Eastern China's high RR, as they directly govern exposure intensity. Landscape and microclimate effects, though significant, operate secondarily by sustaining tick populations and host encounters. Such geographic disparities challenge the utility of uniform warning thresholds and advocate for regionally calibrated risk models. In addition, the limitations of historical data and the need to dynamically update data also need to be considered in risk modeling due to the diverse forms of current climate change.

The observed differences in lag patterns between high and low temperatures/humidity levels reflect biologically plausible mechanisms governing *H. longicornis* behavior and DBV transmission dynamics. The protective effect observed at 0–1.5 months under low-temperature conditions may be related to tick diapause. Tick diapause is a physiologically induced state of suspended development triggered by low temperatures, documented in multiple hard ticks. It delays egg hatching and nymph activity, thereby reducing human exposure opportunities in the short term [49,52]. However, as this study relies on correlation analysis between environmental and case time series, it cannot directly observe changes in the physiological state or life cycle of tick populations. Therefore, this interpretation should be considered a biologically plausible hypothesis.

The study has some limitations. Our findings provide insights into the current associations between meteorological factors and SFTS incidence, but their extrapolation to future climate change scenarios requires careful consideration. Extrapolability is primarily constrained by two factors: first, the study period (e.g., 2011–2023) reflects historical climate conditions, and future shifts in temperature variability (e.g., more frequent extreme heat events) or precipitation patterns (e.g., intensified seasonal rainfall) may alter the observed exposure-response relationships (e.g., the temperature threshold for SFTS risk peaks identified in our analysis). Second, climate change could indirectly modulate SFTS transmission by influencing vector (e.g., *H. longicornis*) ecology (e.g., expanded geographic range, altered biting behavior) or host-virus interactions, which were not explicitly modeled in our current framework. In addition, the lack of surveillance data on ticks and their host animals may allow the independent role of meteorological factors to be overestimated. Further studies are needed to explore the multiple drivers of SFTS.

## 5 Conclusions

Using data from 34 cities (2011−2023), we identified monthly average temperature, precipitation, relative humidity, and atmospheric pressure as key climatic drivers of SFTS with distinct spatial and seasonal patterns. To operationalize these findings within the Smart Multi-Point Triggered Infectious Disease Surveillance and Early Warning System (SMIDSA), it is recommended that regional temperature thresholds (24.7 °C nationwide, 27.5 °C in eastern regions) be embedded into the core algorithm when establishing the SFTS early warning system. Additionally, a 2-month lead time should be incorporated to account for delayed tick development.

For tick management, concentrate monitoring and interventions in the month before and during local SFTS peak months, report tick density, tick infection prevalence and host seroprevalence monthly, and trigger targeted tick control agents, vegetation and livestock management, and community protection campaigns when indicators or SMIDSA action alerts are met. Provinces should calibrate thresholds with 3–5 years of local data and automate data linkage for weekly rule evaluation; evaluate performance by timeliness, peak-month detection rate, and resource efficiency. These steps convert our climate-disease associations into concrete early-warning rules and intervention pathways to enable proactive risk management and reduce SFTS burden.

## CRediT authorship contribution statement

**Guangju Mo:** Writing – original draft, Methodology, Investigation, Formal analysis, Data curation, Conceptualization. **Xiyuan Huo:** Formal analysis, Data curation. **Meshack Kipkogei Biwott:** Writing – review & editing, Formal analysis. **Nan Chang:** Methodology, Formal analysis, Data curation. **Haoqiang Ji:** Methodology, Data curation. **Lianfang Feng:** Methodology, Investigation. **Huaiping Zhu:** Writing – review & editing, Supervision, Conceptualization. **Qiyong Liu:** Writing – review & editing, Supervision, Funding acquisition.

## Consent for publication

Not applicable.

## Ethics approval and consent to participate

Not applicable.

## Funding

This study was funded by grants from consultancy project (2023-JB-12) by the Chinese Academy of Engineering (CAE), the Guangdong Provincial Key Area Research and Development Program (2022B1111030002), Comprehensive Innovation Capability Support of Intelligent Tracking and Forecasting for Infectious Diseases (grant number 102393240020020000004 - 2025NITFID715), and Research Project on the Spread Risks of Known Important Vector Organisms and of Known Important Vector Organisms and Their Carried Pathogens in China.

## Declaration of competing interest

The authors declare that they have no known competing financial interests or personal relationships that could have appeared to influence the work reported in this paper.

## Data Availability

The datasets analyzed during the current study are available from the corresponding author on reasonable request.

## Appendix A Supplementary data

Supplementary information Image 1
