# Supplementary material for: The effect of meteorological factors on severe fever with thrombocytopenia syndrome: Evidence from 34 Chinese cities
Source: One Health. 2025 Dec 11;22:101295. doi: 10.1016/j.onehlt.2025.101295 (PMC12811632; doi:10.1016/j.onehlt.2025.101295)
Supplement: Supplementary file 1 — Supplementary information [file mmc1.docx]

**Supplementary information**

**The effect of meteorological factors on severe fever with thrombocytopenia syndrome: Evidence from 34 Chinese cities**

**Guangju Mo^1,2^, Xiyuan Huo^3^, Meshack Kipkogei Biwott^1^, Nan Chang^4^, Haoqiang Ji^5^, Lianfang Feng^5^, Huaiping Zhu^6*^, Qiyong Liu^1,2*^**

**eMethods.** Definition of SFTS

**eReferences.**

**eTable 1.** Evaluation of Quality of Cross-sectional Studies of Evidence

**eTable 2.** Summary of Total SFTS Cases and Monthly Meteorological Variables in 34 Cities from 2011 to 2023

**eTable 3.** Descriptive Statistics of Monthly Meteorological Factors in 34 Cities, 2011−2023

**eFigure 1.** Sample Inclusion and Exclusion Criteria

**eFigure 2.** Correlation of SFTS Cases with Meteorological, Environmental, and Social Factors, 2011-2023

**eFigure 3.** Lagged Effects of Specific Average Temperature on SFTS Incidence

**eFigure 4.** Lagged Effects of Specific Average Relative Humidity on SFTS Incidence

**eFigure 5.** Lagged Effects of Specific Average Atmospheric Pressure on SFTS Incidence

**eFigure 6.** Lagged Effects of Specific Average Precipitation on SFTS Incidence

**eMethods.** Definition of SFTS

Clinically diagnosed SFTS cases were defined as fever (temperature ≥ 38℃) accompanied by either thrombocytopenia, or hemorrhagic symptoms (at least one of the hemorrhagic symptoms such as dark stools, gingival bleeding, skin petechiae or ecchymoses, and conjunctival congestion). Suspected cases were defined as individuals with a history of working, living, or traveling in hilly, forested, or mountainous areas during the epidemic season, or a history of tick bites within 2 weeks before the onset of illness, clinical signs such as fever, and decreased peripheral blood platelets and white blood cells. A confirmed case was defined as a suspected case meeting at least one of the following laboratory criteria: (a) case specimen positive for SFTS virus nucleic acid; (b) case specimen positive for SFTS virus IgG antibody or recovery phase titer more than four times higher than the acute phase; and (c) isolation of SFTS virus from case specimen^1,2^.

**eReferences.**

1. Liu Q, He B, Huang SY, Wei F, Zhu XQ. Severe fever with thrombocytopenia syndrome, an emerging tick-borne zoonosis. *Lancet Infect Dis.* 2014;14(8):763-772. doi:10.1016/S1473-3099(14)70718-2
2. Xie YT, Lai DH, Liu GY, Zhou JL, Lun ZR. Severe fever with thrombocytopenia syndrome in China.*Lancet Infect Dis*. 2015;15(2):145. doi:10.1016/S1473-3099(14)70891-6

**eTable 1.** Evaluation of Quality of Cross-sectional Studies of Evidence

|  | Item No | Recommendation | Page No |
| --- | --- | --- | --- |
| **Title and abstract** | 1 | (*a*) Indicate the study’s design with a commonly used term in the title or the abstract | 1 |
|  |  | (*b*) Provide in the abstract an informative and balanced summary of what was done and what was found | 1-2 |
| Introduction | | | |
| Background/rationale | 2 | Explain the scientific background and rationale for the investigation being reported | 3-4 |
| Objectives | 3 | State specific objectives, including any prespecified hypotheses | 4-5 |
| Methods | | | |
| Study design | 4 | Present key elements of study design early in the paper | 6 |
| Setting | 5 | Describe the setting, locations, and relevant dates, including periods of recruitment, exposure, follow-up, and data collection | 6-7 |
| Participants | 6 | (*a*) Give the eligibility criteria, and the sources and methods of selection of participants | 6-7 |
| Variables | 7 | Clearly define all outcomes, exposures, predictors, potential confounders, and effect modifiers. Give diagnostic criteria, if applicable | 6-7 |
| Data sources/ measurement | 8* | For each variable of interest, give sources of data and details of methods of assessment (measurement). Describe comparability of assessment methods if there is more than one group | 6-7 |
| Bias | 9 | Describe any efforts to address potential sources of bias | 8 |
| Study size | 10 | Explain how the study size was arrived at | NA |
| Quantitative variables | 11 | Explain how quantitative variables were handled in the analyses. If applicable, describe which groupings were chosen and why | 8-9 |
| Statistical methods | 12 | (*a*) Describe all statistical methods, including those used to control for confounding | 7-9 |
|  |  | (*b*) Describe any methods used to examine subgroups and interactions | 7-9 |
|  |  | (*c*) Explain how missing data were addressed | NA |
|  |  | (*d*) If applicable, describe analytical methods taking account of sampling strategy | NA |
|  |  | (*e*) Describe any sensitivity analyses | 9 |
| Results | | | |
| Participants | 13* | (a) Report numbers of individuals at each stage of study—eg numbers potentially eligible, examined for eligibility, confirmed eligible, included in the study, completing follow-up, and analysed | 10 |
|  |  | (b) Give reasons for non-participation at each stage | 10 |
|  |  | (c) Consider use of a flow diagram | Page 8 in Supplementary file |
| Descriptive data | 14* | (a) Give characteristics of study participants (eg demographic, clinical, social) and information on exposures and potential confounders | Page 6 in Supplementary file |
|  |  | (b) Indicate number of participants with missing data for each variable of interest | NA |
| Outcome data | 15* | Report numbers of outcome events or summary measures | 9 |
| Main results | 16 | (*a*) Give unadjusted estimates and, if applicable, confounder-adjusted estimates and their precision (eg, 95% confidence interval). Make clear which confounders were adjusted for and why they were included | 10-13 |
|  |  | (*b*) Report category boundaries when continuous variables were categorized | Page 7 in Supplementary file |
|  |  | (*c*) If relevant, consider translating estimates of relative risk into absolute risk for a meaningful time period | NA |
| Other analyses | 17 | Report other analyses done—eg analyses of subgroups and interactions, and sensitivity analyses | 16-18 |
| Discussion | | | |
| Key results | 18 | Summarise key results with reference to study objectives | 18 |
| Limitations | 19 | Discuss limitations of the study, taking into account sources of potential bias or imprecision. Discuss both direction and magnitude of any potential bias | 21-22 |
| Interpretation | 20 | Give a cautious overall interpretation of results considering objectives, limitations, multiplicity of analyses, results from similar studies, and other relevant evidence | 18-21 |
| Generalisability | 21 | Discuss the generalisability (external validity) of the study results | 21 |
| Other information | | | |
| Funding | 22 | Give the source of funding and the role of the funders for the present study and, if applicable, for the original study on which the present article is based | 25 |

*Give information separately for exposed and unexposed groups.

**eTable 2.** Summary of total SFTS cases and monthly meteorological variables in 34 cities from 2011 to 2023

| City | Total SFTS cases | Monthly average | | | | |
| --- | --- | --- | --- | --- | --- | --- |
|  |  | Temperature (℃) | Relative humidity (%) | Atmospheric pressure (hPa) | Wind speed (m/s) | Precipitation (mm) |
| Xinyang | 5999 | 16.29 | 70.39 | 1004.65 | 0.76 | 3.01 |
| Huanggang | 1937 | 16.76 | 72.85 | 992.56 | 0.49 | 4.05 |
| Yantai | 1924 | 13.04 | 62.97 | 1003.31 | 0.95 | 1.94 |
| Chuzhou | 1501 | 16.15 | 71.99 | 1010.76 | 1.22 | 3.06 |
| Weihai | 1431 | 12.55 | 66.76 | 1008.07 | 0.92 | 1.99 |
| Jinan | 1269 | 13.81 | 61.98 | 1002.70 | 0.69 | 2.13 |
| Lu’an | 1133 | 15.99 | 72.60 | 996.02 | 0.79 | 3.51 |
| Hefei | 1117 | 16.76 | 72.54 | 1011.45 | 1.14 | 3.34 |
| Suizhou | 1104 | 16.13 | 70.75 | 994.92 | 0.57 | 2.85 |
| Anqing | 859 | 16.26 | 74.37 | 984.92 | 0.71 | 4.64 |
| Dalian | 697 | 9.93 | 65.36 | 998.02 | 0.98 | 2.10 |
| Tai'an | 687 | 14.06 | 62.09 | 994.48 | 0.57 | 2.29 |
| Linyi | 628 | 14.37 | 62.18 | 994.42 | 0.86 | 2.40 |
| Dandong | 558 | 7.50 | 67.59 | 978.27 | 0.47 | 2.88 |
| Nanjing | 504 | 16.59 | 71.79 | 1013.43 | 1.27 | 3.49 |
| Qingdao | 493 | 13.58 | 62.80 | 1009.88 | 0.82 | 2.05 |
| Weifang | 480 | 13.91 | 60.56 | 1005.27 | 0.82 | 2.01 |
| Ma’anshan | 420 | 16.81 | 72.89 | 1012.21 | 1.33 | 3.62 |
| Zibo | 367 | 13.73 | 59.76 | 990.80 | 0.76 | 2.21 |
| Xiaogan | 331 | 16.70 | 72.96 | 1002.41 | 0.45 | 3.29 |
| Taizhou | 293 | 16.73 | 77.50 | 978.78 | 0.54 | 4.63 |
| Rizhao | 276 | 13.89 | 62.59 | 996.62 | 0.69 | 2.26 |
| Huai’an | 253 | 15.70 | 71.14 | 1015.18 | 1.10 | 2.86 |
| Nanyang | 237 | 15.02 | 66.50 | 970.88 | 0.69 | 2.31 |
| Xuancheng | 191 | 16.57 | 75.77 | 998.43 | 0.75 | 4.76 |
| Zhoushan | 172 | 17.58 | 77.22 | 1013.63 | 1.15 | 4.32 |
| Shaoxing | 162 | 17.17 | 75.97 | 1000.37 | 0.62 | 4.63 |
| Wuhan | 145 | 17.49 | 72.84 | 1009.32 | 0.61 | 3.74 |
| Dezhou | 141 | 13.81 | 62.73 | 1014.18 | 0.78 | 1.81 |
| Xianning | 139 | 17.66 | 74.65 | 1002.53 | 0.55 | 4.27 |
| Tongling | 122 | 17.06 | 74.61 | 1009.91 | 0.80 | 4.16 |
| Chizhou | 118 | 17.03 | 76.40 | 1004.32 | 0.61 | 5.05 |
| Jinhua | 113 | 17.28 | 73.98 | 986.89 | 0.59 | 4.32 |
| Ningbo | 100 | 17.09 | 76.11 | 993.61 | 0.76 | 4.62 |

**eTable3.** Descriptive statistics of monthly meteorological factors in 34 cities, 2011−2023

| Meteorological factors | Min | P_5_ | P_50_ | P_95_ | Max |
| --- | --- | --- | --- | --- | --- |
| monthly average temperature (℃) | -15.45 | 0.01 | 16.39 | 28.22 | 31.49 |
| monthly average relative humidity (%) | 33.26 | 50.47 | 71.35 | 84.04 | 95.24 |
| monthly average atmospheric pressure (hPa) | 944.24 | 976.35 | 1000.94 | 1021.40 | 1030.35 |
| monthly average wind speed (m/s) | 0.16 | 0.39 | 0.72 | 1.39 | 2.08 |
| monthly average precipitation (mm) | 0.00 | 0.14 | 2.38 | 9.17 | 26.67 |

Note: P_5_ is the 5th percentile, P_50_ is the 50th percentile, and P_95_ is the 95th percentile.

**eFigure 1.** Sample Inclusion and Exclusion Criteria


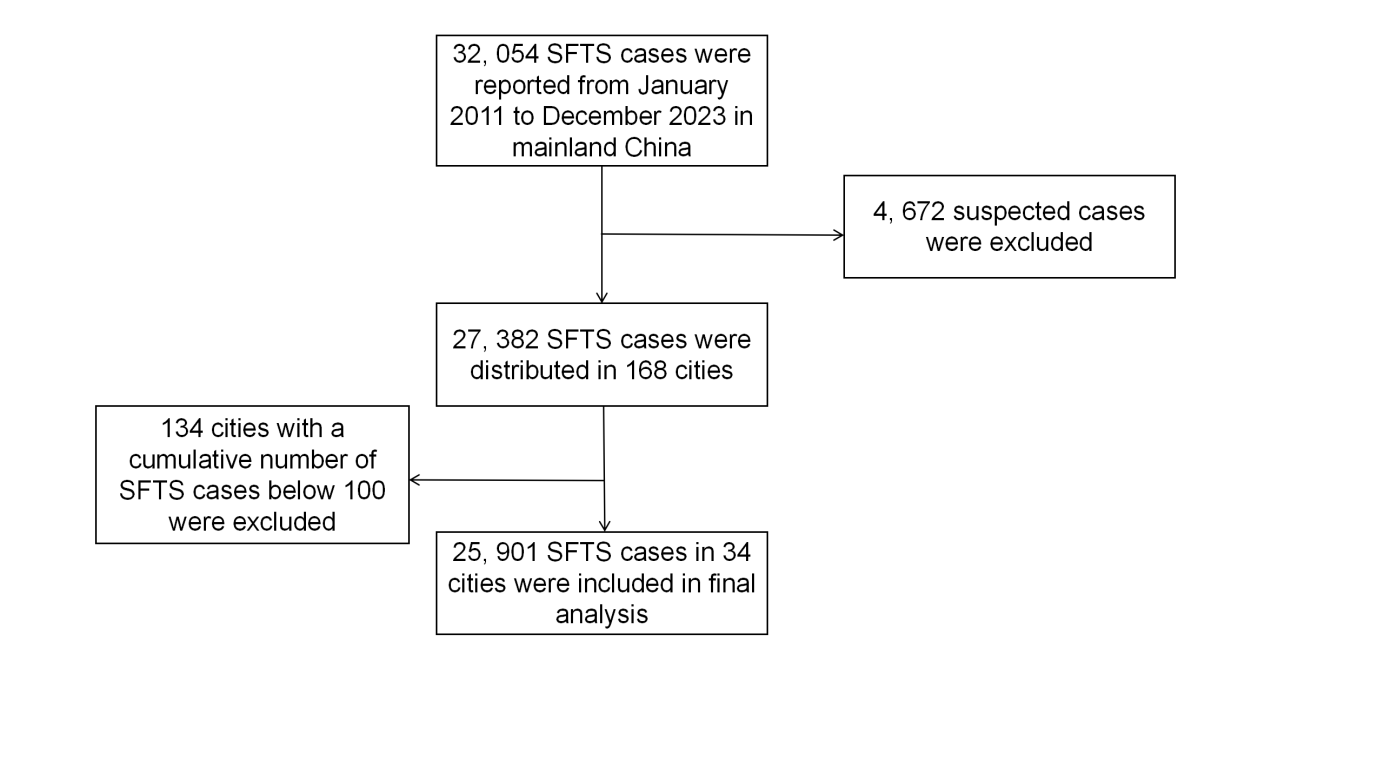


**eFigure 1.** Sample Inclusion and Exclusion Criteria

**eFigure 2.** Correlation of SFTS Cases with Meteorological, Environmental, and Social Factors, 2011-2023

**
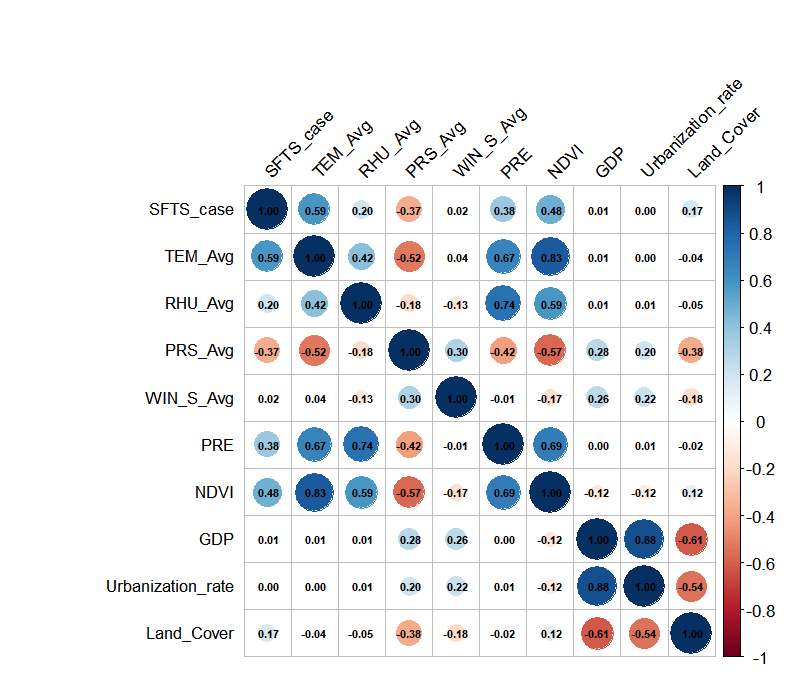
**

**eFigure 2.** Correlation of SFTS Cases with Meteorological, Environmental, and Social Factors, 2011-2023

**eFigure 3.** Lagged Effects of Specific Average Temperature on SFTS Incidence

**
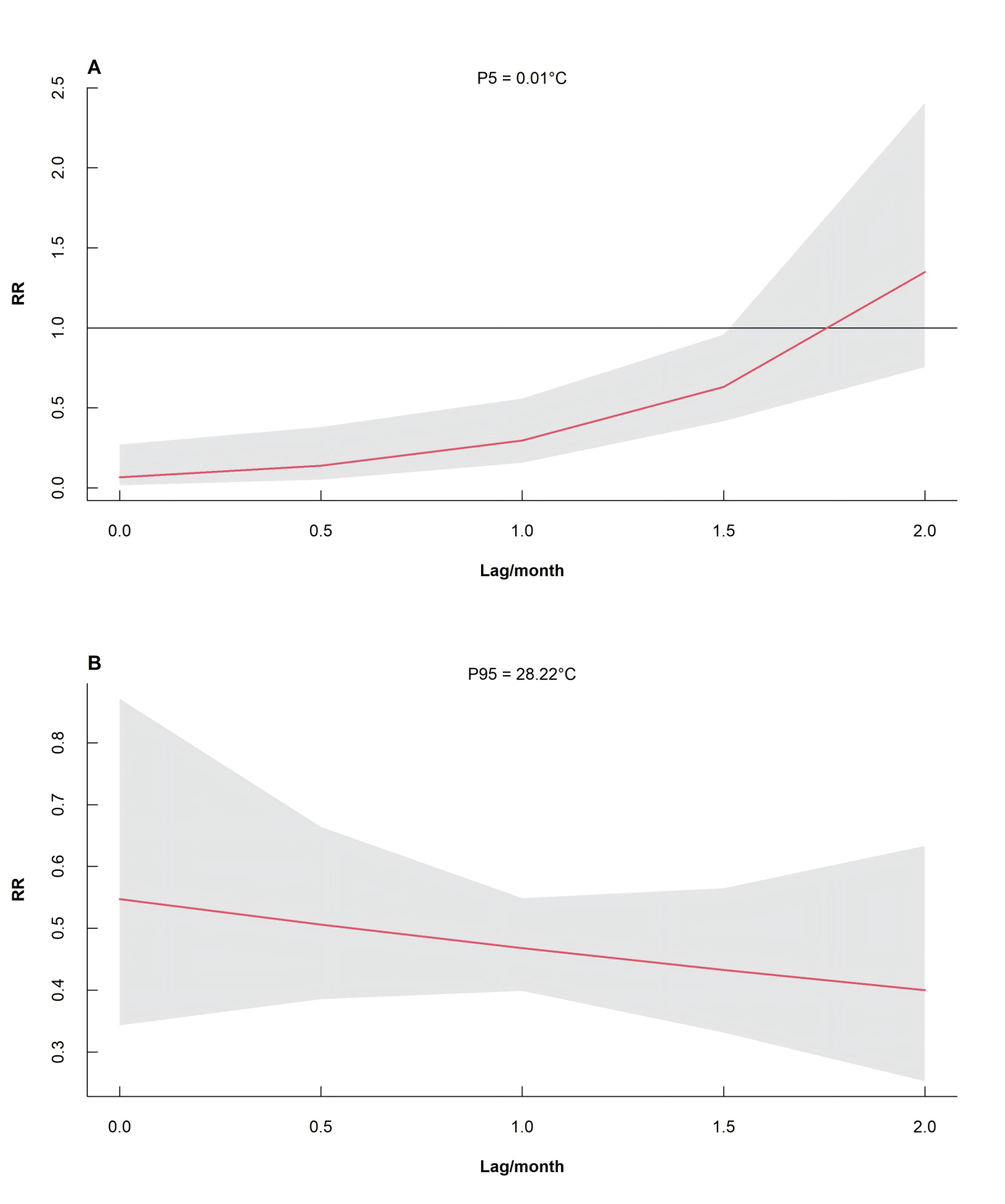
**

**eFigure 3.** Lagged Effects of Specific Average Temperature on SFTS Incidence

**eFigure 4.** Lagged Effects of Specific Average Relative Humidity on SFTS Incidence

**
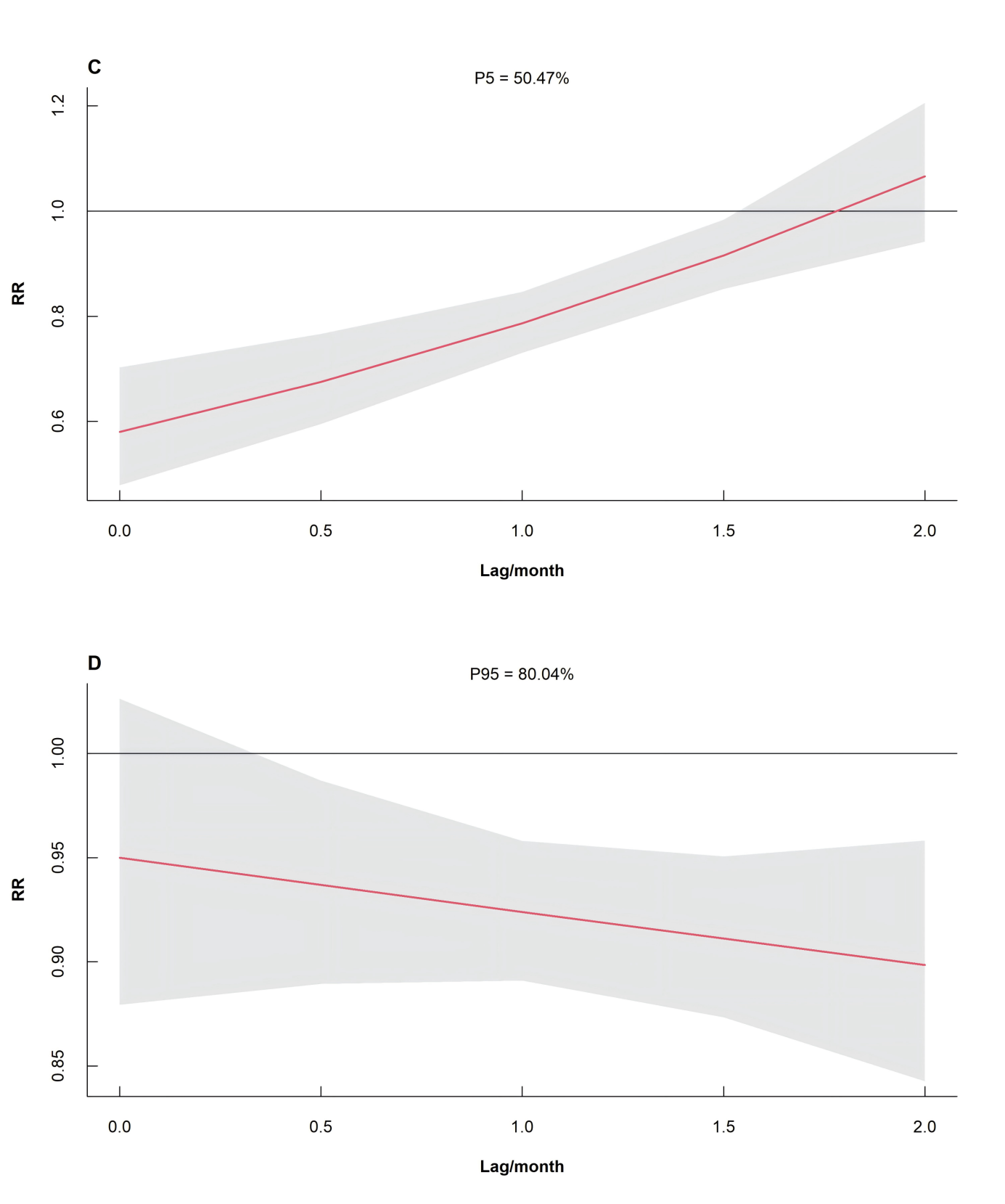
**

**eFigure 4.** Lagged Effects of Specific Average Relative Humidity on SFTS Incidence

**eFigure 5.** Lagged Effects of Specific Average Atmospheric Pressure on SFTS Incidence

**
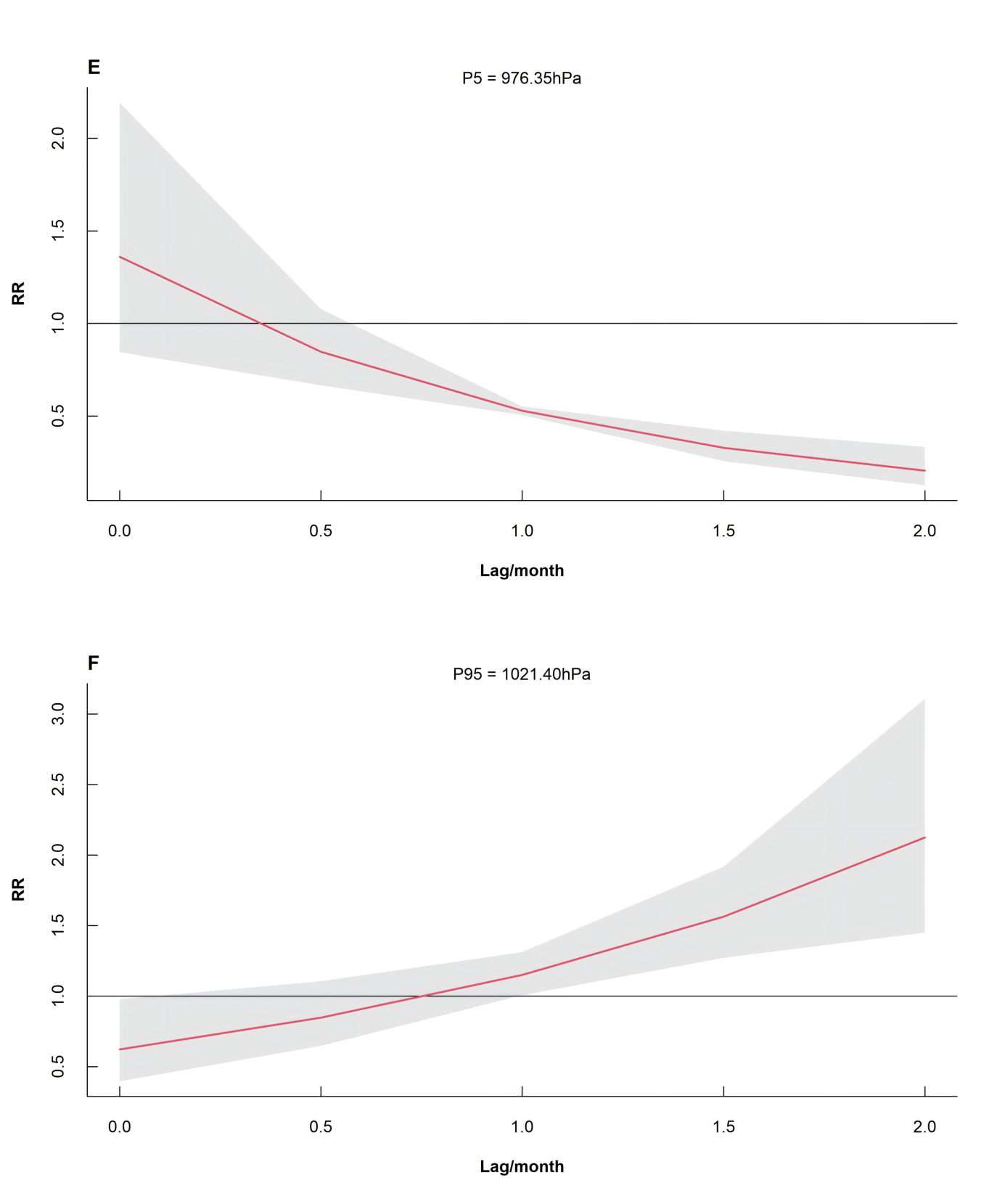
**

**eFigure 5.** Lagged Effects of Specific Average Atmospheric Pressure on SFTS Incidence

**eFigure 6.** Lagged Effects of Specific Average Precipitation on SFTS Incidence

**
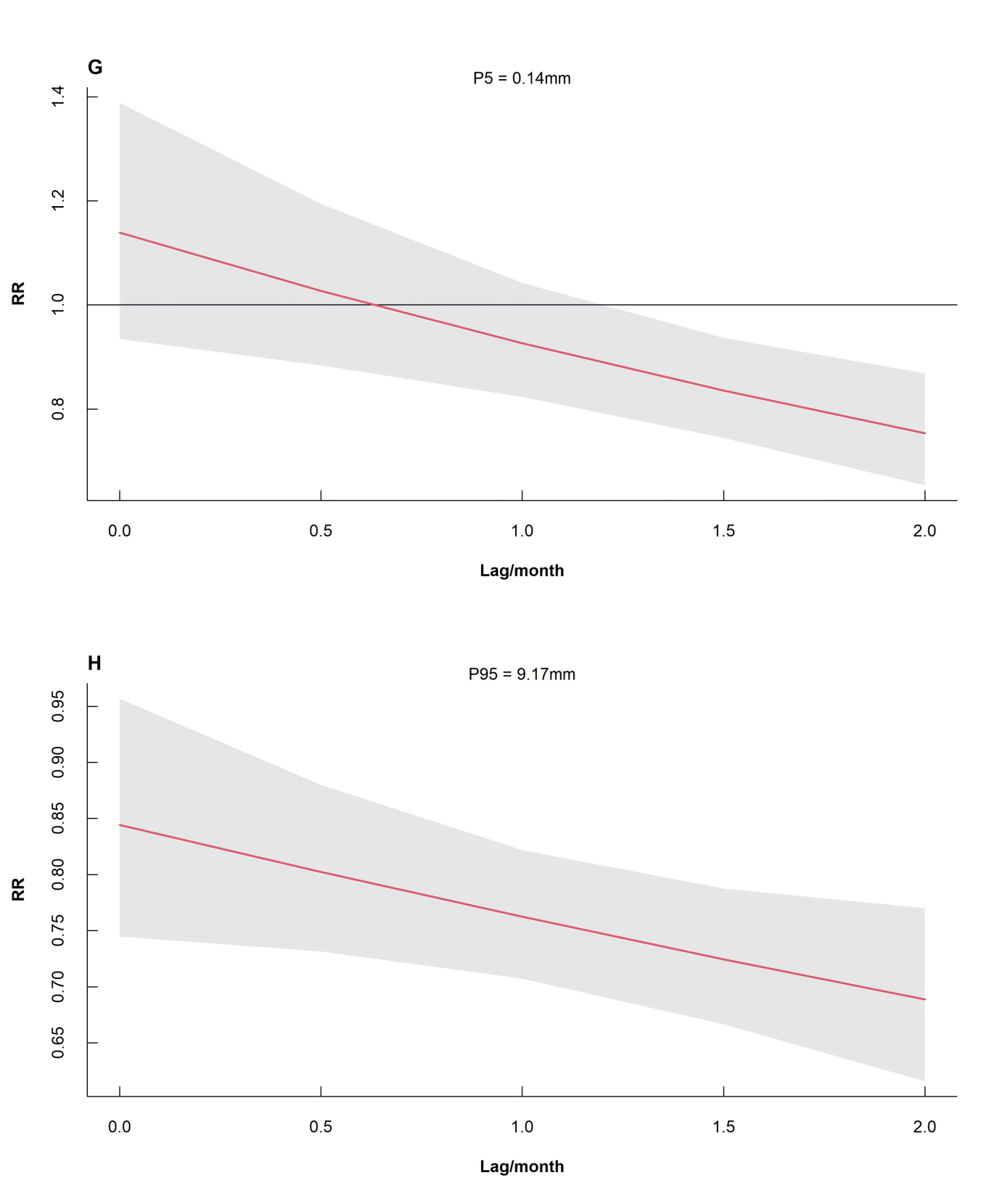
**

**eFigure 6.** Lagged Effects of Specific Average Precipitation on SFTS Incidence .
